# Sustained and intermittent hypoxia differentially modulate primary monocyte immunothrombotic responses to IL-1β stimulation

**DOI:** 10.3389/fimmu.2023.1240597

**Published:** 2023-09-11

**Authors:** Casper J.E. Wahlund, Safak Çaglayan, Paulo Czarnewski, John-Bjarne Hansen, Omri Snir

**Affiliations:** ^1^ Thrombosis Research Group (TREC), Department of Clinical Medicine, UiT – The Arctic University of Norway, Tromsø, Norway; ^2^ Science for Life Laboratory, Department of Gene Technology, KTH Royal Institute of Technology, Stockholm, Sweden; ^3^ Science for Life Laboratory, Department of Biochemistry and Biophysics, National Bioinformatics Infrastructure Sweden, Stockholm University, Stockholm, Sweden; ^4^ Division of Internal Medicine, University Hospital of North Norway, Tromsø, Norway

**Keywords:** monocytes, venous thromboembolism, immunothrombosis, tissue factor, SPP1, deep vein thrombosis, intermittent hypoxia

## Abstract

Venous thromboembolism (VTE) is a leading cause of preventable deaths in hospitals, and its incidence is not decreasing despite extensive efforts in clinical and laboratory research. Venous thrombi are primarily formed in the valve pockets of deep veins, where activated monocytes play a crucial role in bridging innate immune activation and hemostatic pathways through the production of inflammatory cytokines, chemokines, and tissue factor (TF) – a principal initiator of coagulation. In the valve pocket inflammation and hypoxia (sustained/intermittent) coexist, however their combined effects on immunothrombotic processes are poorly understood. Inflammation is strongly associated with VTE, while the additional contribution of hypoxia remains largely unexplored. To investigate this, we modelled the intricate conditions of the venous valve pocket using a state-of-the-art hypoxia chamber with software-controlled oxygen cycling. We comprehensively studied the effects of sustained and intermittent hypoxia alone, and in combination with VTE-associated inflammatory stimuli on primary monocytes. TF expression and activity was measured in monocytes subjected to sustained and intermittent hypoxia alone, or in combination with IL-1β. Monocyte responses were further analyzed in detailed by RNA sequencing and validated by ELISA. Stimulation with IL-1β alone promoted both transcription and activity of TF. Interestingly, the stimulatory effect of IL-1β on TF was attenuated by sustained hypoxia, but not by intermittent hypoxia. Our transcriptome analysis further confirmed that sustained hypoxia limited the pro-inflammatory response induced by IL-1β, and triggered a metabolic shift in monocytes. Intermittent hypoxia alone had a modest effect on monocyte transcript. However, in combination with IL-1β intermittent hypoxia significantly altered the expression of 2207 genes and enhanced the IL-1β-stimulatory effects on several chemokine and interleukin genes (e.g., IL-19, IL-24, IL-32, MIF), as well as genes involved in coagulation (thrombomodulin) and fibrinolysis (VEGFA, MMP9, MMP14 and PAI-1). Increased production of CCL2, IL-6 and TNF following stimulation with intermittent hypoxia and IL-1β was confirmed by ELISA. Our findings provide valuable insights into how the different hypoxic profiles shape the immunothrombotic response of monocytes and shed new light on the early events in the pathogenesis of venous thrombosis.

## Introduction

Innate immune responses to invading pathogens involve inflammation-induced local coagulation to limit the ability of pathogens to spread (1). This process, which is mediated primarily in the micro vessels, is a potent line of defense and the basis for the concept of ‘Immunothrombosis’ (2). However, if the tightly controlled cross-talk between innate immune cells and the coagulation system is dysregulated, the consequence may be life-threatening thrombosis (2). Monocytes are potent producers of chemokines, and interleukins (3) including IL-6 and IL-8 (4), drive immunothrombosis, and promote activation of platelets, neutrophils and endothelial cells (1, 3). Of particular note, activated monocytes are the main source of intravascular tissue factor (TF), the primary activator of the coagulation system.

Venous thromboembolism (VTE), encompassing deep vein thrombosis and pulmonary embolism, affects more than 10 million individuals annually worldwide (5). Despite extensive mapping of risk factors for VTE, about half of the patients have minor or no known risk factors (6, 7). Moreover, the sequence of events leading to VTE is largely unclear, which Cushman et al. recently identified as a top research priority of VTE (8). The lack of detailed understanding how and why VTE is initiated hinders the development of targeted prevention and treatment strategies for the disease. It is therefore critical to address these knowledge gaps and to uncover the pathophysiological mechanisms in VTE, including the role of immunothrombotic processes (8). Importantly, venous thrombi predominantly form in valve pockets of the deep veins, an anatomical site characterized by turbulent flow-of-blood and strongly alternating oxygenation, i.e., *intermittent hypoxia*. Such conditions may trigger and drive local inflammation in the venous valve, which has been suggested to promote thrombosis (9) but direct evidence of this is mostly lacking. The potential role for hypoxia in the pathophysiology of VTE has intrigued researchers for decades. Observational studies indicate that high altitude stay is accompanied by an elevated risk of VTE, but it remains unclear whether this is solely due to oxygen deficiency and through which mechanisms hypoxia would promote thrombosis (10). Early *in vivo* experiments conducted in the 1980s showed that the oxygen levels in the valve pocket drop to nearly zero during stasis (11), and that periods of stasis followed by intense muscular movements lead to intermittent hypoxia and increased thrombus formation (12). Intermittent hypoxia is also associated with other cardiovascular diseases including myocardial infarction, acute coronary syndrome, sepsis, and organ transplantation (13, 14). Periodic hypoxic stress in the veins is however common and neither stasis nor hypoxia alone is sufficient to promote thrombosis (15). It is more likely that VTE initiation involves a combination of factors that collectively lower the threshold for thrombogenesis, but whether hypoxia and inflammation act together in VTE remains uncertain (15).

In the current study we investigate how primary monocyte function is affected in a simulated pro-thrombotic environment resembling that of the venous valve pockets. To achieve this, we combined hypoxia and inflammatory IL-1β stimulation. Additionally, we address the importance of the specific hypoxic pattern (sustained vs intermittent) in relation to the immunothrombotic response. To the best of our knowledge, this is the first study to systematically explore both the individual and the combined effects of sustained and intermittent hypoxia, along with IL-1β stimulation, on the monocyte transcriptome and cytokine release.

## Materials and methods

### Donors and blood sampling

Peripheral venous blood was collected after overnight fasting from nine healthy donors (five women and four men), who gave a written informed consent. The blood donors were on average 43 years old (27-61). Blood counts were within normal ranges for leukocytes, erythrocytes, and platelets. All donor recruitment and handling of data and samples was conducted in line with the Helsinki declaration and an ethical permit for the study approved by a regional ethics committee of northern Norway. The samples were anonymized, only age and sex were registered. Approximately 50 ml was collected in vacuum tubes prefilled with sodium citrate.

### Monocyte isolations

The collected blood was diluted with an equal volume of calcium-free PBS and transferred into leukosep tubes (Greiner Bio-One, Germany). Peripheral blood mononuclear cells (PBMC) were then separated by density gradient (Lymphoprep, Stemcell Technologies, Canada) according to the manufacturer’s protocol. After washing and counting the PBMCs, monocytes were isolated using a negative selection magnetic bead-based kit for monocytes with magnetic columns (Miltenyi Biotech, Germany). Monocytes were isolated according to the manufacturer’s protocol, with an additional step using CD61-microbeads (Miltenyi Biotech, Germany) to deplete platelets from the cell suspension. Successful cell isolations were confirmed by microscopy and monocyte purity was analyzed by flow cytometry using a Cytoflex flow cytometer (Beckman-Coulter, USA). Initial forward/side scatter gate was set to remove noise, followed by gating on CD14-positive events (antibodies from Biolegend, USA) to assess percentage of CD14^+^ monocytes, showing a purity of 92.8% (89-96%).

### Hypoxic and inflammatory stimulations of monocytes

**Figure 1:**
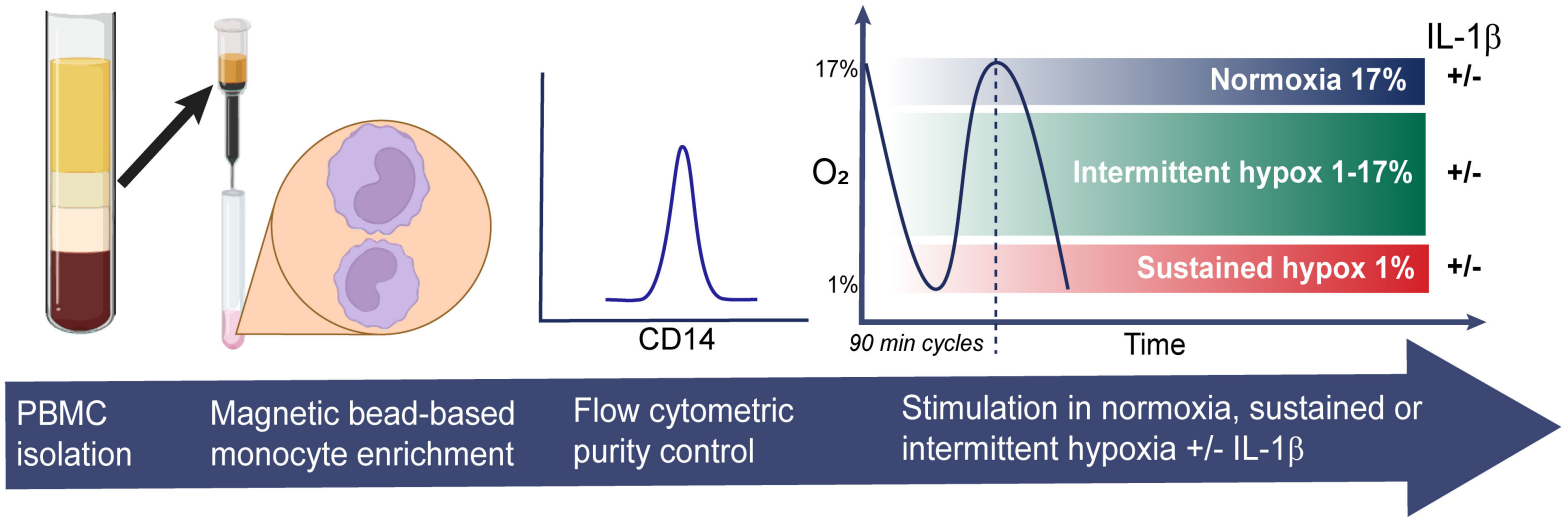
Experimental design. Monocytes were isolated from freshly prepared PBMCs from nine healthy donors using magnetic bead-based negative selection. CD14-purity was assessed by flow cytometry before the monocytes were stimulated in 96-well plates for 4.5 hours in normoxia, sustained or intermittent hypoxia, in presence or absence of IL-1β.

The levels of O_2_ in “normoxic” cell cultures are often incorrectly reported to be that of the surrounding air, approximately 21%, which does not take into account the oxygen-displacing effect of humidity in cell incubators which lower the O_2_ to approximately 18.6% (16). In reality, this number may be even lower depending on the local conditions including ambient air O_2_ levels, how frequently the incubator is used and opened, and the presence of (oxygen-consuming) cells. We therefore used a calibrated oxygen-sensing probe connected to a Fibox 4 gas analyzer (PreSens, Germany) to measure the O_2_ levels in our normoxic cell incubator, which was found to be 17% O_2_. We next set the hypoxic conditions using an Xvivo Systems hypoxia chamber with a glove access compartment (BioSpherix, USA) with a constant CO_2_ level of 5%, with nitrogen as an inert gas balance. We then assessed the gas exchange rate in the cell culture media after inducing cycles of intermittent hypoxia shifting from 17% to 1% O_2_ in the air. Approximately 25 minutes were required for the culture media to shift from normoxic (17% O_2_) to fully hypoxic (1% O_2_). Each period of intermittent hypoxia was therefore set to 45 minutes (consequently 90 minutes for a full cycle) to allow for the cells to be 20 minutes in fully hypoxic media (1% O_2_) before returning to normoxia (17% O_2_). In total the cells were stimulated for 4.5 hours, divided in three cycles (90 minutes each) of intermittent hypoxia. Purified and viable monocytes were plated in 96-well plates with gas-permeable bottoms (Zellkontakt, Germany), 80,000 per well. The cells were kept in RPMI with Glutamax (Gibco) and 5% FBS (Gibco). The cells were allowed to settle for 20 minutes before being stimulated either in normoxia, sustained or intermittent hypoxia, in the absence or the presence of 10 ng/ml IL-1β (Sigma). After 4.5 hours of stimulations, the plates were removed from the incubators and kept on ice while the cells were harvested. Monocytes collected from seven wells (560k) were pooled for RNA isolations; others (160k) were pooled and used for TF activity assays. Cell culture supernatants were centrifuged at 3000xg for 10 minutes and kept in -80° C for analyses of chemokines and cytokines. The cell pellets designated for RNA analysis were resuspended in ice-cold PBS. The cell pellets to be used in the TF activity assays were resuspended in ice-cold HEPES/NaCl and immediately frozen in -80° C. The experimental settings are summarized in **Figure 1**.

### RNA isolation and RT-qPCR

Monocytes resuspended in PBS were spun down, resuspended in QIAzol lysis reagent (Qiagen, USA) and froze at -80° C to preserve the RNA before further isolation. Total RNA was then isolated using a miRNeasy kit according to manufacturer’s protocol (Qiagen, USA). An aliquot of the RNA was reverse transcribed using a miRCURY RT kit (Qiagen, USA) for qPCR analyses of Tissue Factor (*F3*). The F3 gene expression was normalized to two housekeeping genes: actin B (ACTB) and TATA-box binding protein (TBP) using the 2−ΔΔCT method (17) and presented as relative fold change in comparison to normoxia control.

### RNA sequencing

RNA yield and integrity were assessed using Nanodrop (Thermo Scientific, USA), and RNA 6000 Nano kit (Agilent Technologies, USA), showing an average RIN value of 9.5 (8.3-10). For most efficient use of resources, and to reduce the impact of individual donor heterogeneity, the RNA from the treated cells of the nine donors was combined into three pools with RNA from three donors each. Libraries of cDNA were prepared by TruSeq RNA library kit (Illumina) according to the manufacturer’s instructions and sequenced using a NovaSeq 6000 at the Norwegian Sequencing Center, Oslo. Reads were pseudomapped to the human transcriptome (GRCh38.p13) using Salmon v1.5.1. Counts were combined and imported into R for downstream data analysis. Transcripts were summed to gene level and filtered if 5 or less counts were detected in 3 samples, or fewer. Patient-level batch effects were corrected using ComBat implemented in the sva package (18). Differential gene expression was carried out in EdgeR (19) using interaction between hypoxic condition and stimulant, where genes with fold change above 1.5 and p-value below 0.001 were considered significant. Selected genes were grouped into gene modules of similar expression by hierarchical clustering on correlation distance and “ward.D2” linkage, which were further used for gene set analysis using enrichR (20).

### Tissue factor activity assay

Monocyte TF activity was assessed using a chromogenic assay recently developed in our lab (21). In brief, aliquots corresponding to approximately 25,000 lysed monocytes (control, or stimulated with IL-1β under normoxia, sustained and intermittent hypoxia) were transferred to a 96-well plate in duplicates. A standard dilution of TF (Innovin) was added to each plate. A pre-warmed cocktail of coagulation factors FII, FX, FVIIa, FV/Va (all from Enzyme laboratories, USA) was added to the plate and incubated for 5 minutes at 37° C. Next, pre-warmed 50 mM CaCl2 (15 µl) in 20 mM HEPES/150 mM NaCl buffer was added, followed by an addition of Thrombin chromogenic substrate (Sekisui Diagnostics). An acidic stopping buffer was added after 5 minutes, and the optical density was read at 405 nm a MultiScan FC plate reader (Thermo Fisher Scientific). TF activity was calculated based on the Innovin standard curve fitted with a third order polynomial and presented in fM.

### ELISA

The concentration of CCL2, TNF and IL-6 in monocyte culture supernatants were determined using ELISA (Invitrogen). Supernatants were concentrated 3x using spin filter columns (Pierce, Thermo Scientific) and samples were tested according to manufacturer’s protocol in duplicates which were averaged for absolute concentration calculations.

### Data and code availability

All sequencing data produced herein was deposited on GEO with the accession number GSE234701, except for the raw RNA sequences that due to Norwegian laws on GDRP may not be shared publicly. The raw data is instead deposited in the secure Norwegian federated European Genome Archive (EGA).

### Figures and statistics

Several figures were partly designed in BioRender. Data from the RT-qPCR, TF activity assays, and ELISAs were analyzed using Graph Pad Prism v9. Based on normality tests, the qPCR data was analyzed using Kruskal-Wallis test with Dunn’s *post hoc* adjustments, and the TF activity and ELISA data were analyzed using one-way Anova with Tukey’s *Post hoc* test.

## Results

### Inflammation and hypoxia regulate monocytic tissue factor expression and activity

**Figure 2:**
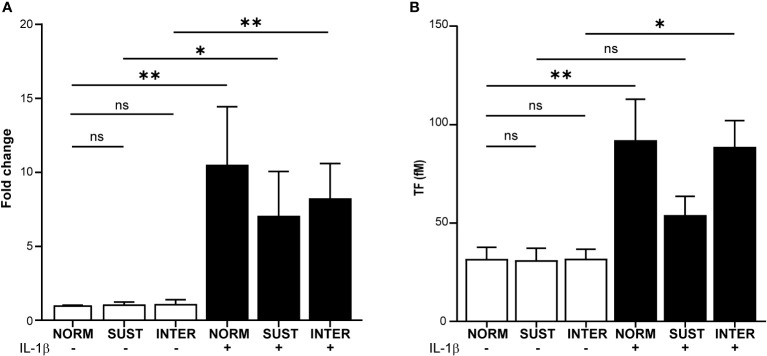
Sustained hypoxia limits the IL-1β induced tissue factor gene expression and TF activity in primary monocytes. Primary monocytes were cultured for 4.5 hours in normoxia (NORM), sustained (SUST) or intermittent (INTER) hypoxia in the absence or presence of IL-1β. **(A)** RT-qPCR analysis of monocytic tissue factor (TF) expression normalized to housekeeping genes and presented as a fold change in comparison to unstimulated monocyte in normoxia (control). **(B)** TF activity measured in the lysates of stimulated monocytes. *p<0.05, **p<0.01, ns = non-significant. Kruskal-Wallis with Dunn’s *post hoc* test for the RT-qPCR TF expression analysis and Ordinary one-way Anova with Tukey’s *Post hoc* test for the TF activity assay.

Monocytes are the main intravascular source of TF, which is a central immunothrombotic driver. We therefore evaluated the impact of VTE-associated pathophysiological stimuli on monocyte TF gene expression and TF activity. Freshly isolated primary monocytes were exposed to sustained hypoxia, intermittent hypoxia or IL-1β alone. As a model for combined inflammatory and hypoxic stress, monocytes were also stimulated with IL-1β in sustained or intermittent hypoxia. Following stimulation, the expression of TF was monitored by RT-qPCR. Neither sustained nor intermittent hypoxia alone affected the expression of TF mRNA (**Figure 2A**). Stimulation of monocytes in normoxia with IL-1β increased the expression of TF by 10.6-fold (p=0.003). A 7.1-fold increased TF expression was monitored when monocytes were stimulated with IL-1β in sustained hypoxia (p=0.04), and 8.3-fold in intermittent hypoxia (p=0.002). Next, we assessed the effects on monocyte TF activity by sustained/intermittent hypoxia and IL-1β. As shown in **Figure 2B**, TF activity under the different stimulatory conditions followed a similar pattern as the mRNA expressions. Neither sustained nor intermittent hypoxia alone had a direct effect on TF activity. IL-1β induced the activity of monocytic TF in normoxia (p=0.009) and in intermittent hypoxia (p=0.016), but not in sustained hypoxia (**Figure 2B**).

### Transcriptome analysis of monocytes subjected to different hypoxic profiles and inflammatory stimulation

**Figure 3:**
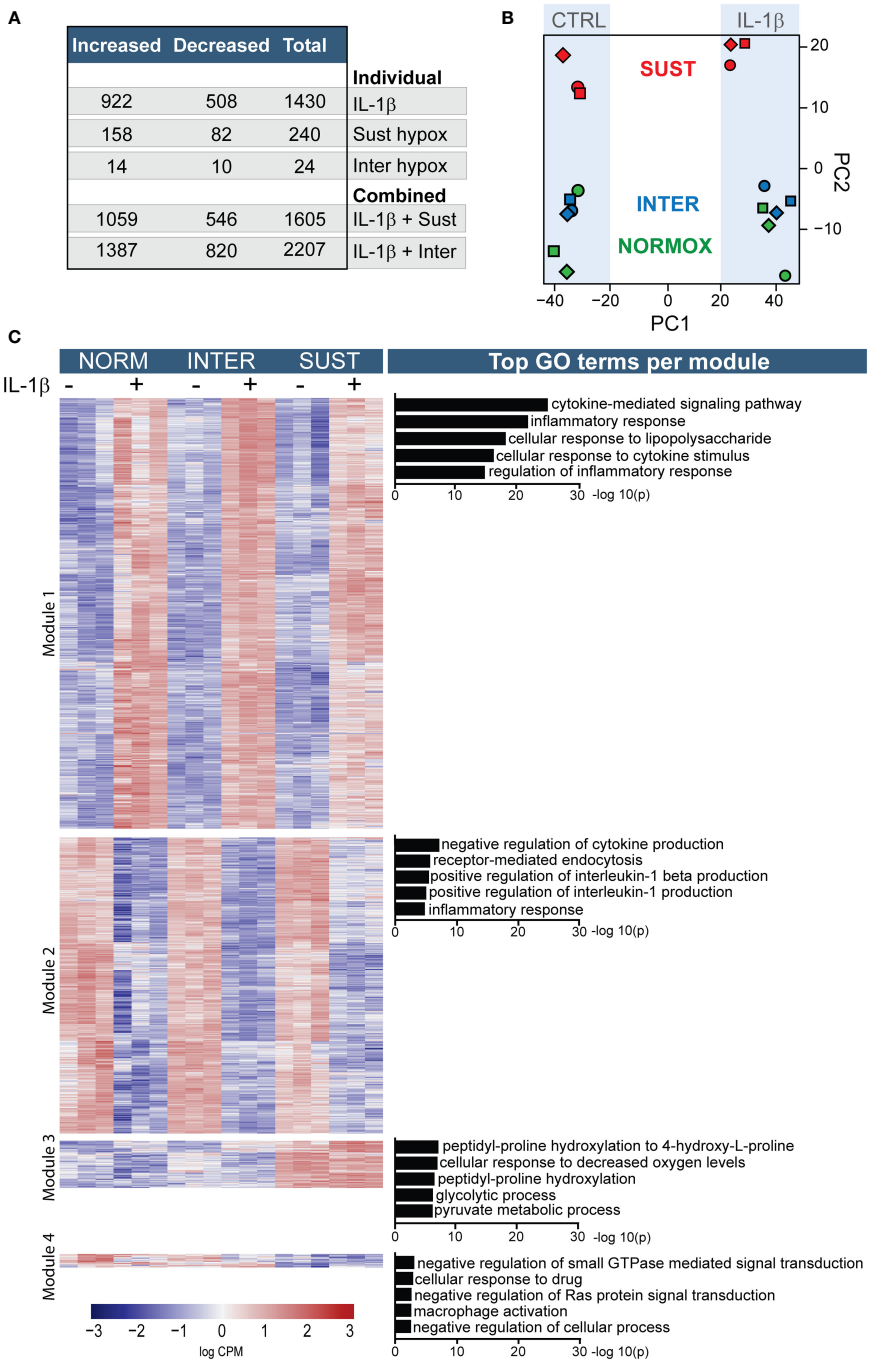
Sustained and intermittent hypoxia differentially modulate the transcriptomic responses to IL-1β stimulation. Primary monocytes from nine different donors were subjected to normoxia (NORM), sustained (SUST) or intermittent (INTER) hypoxia in absence or presence of IL-1β for 4.5 hours. Monocyte transcripts were analyzed in pools of three donors each using RNA sequencing. **(A)** Overview of the differential gene expression counts in comparison to unstimulated monocytes in normoxia. **(B)** Principal component analysis of the monocyte transcriptome in the six tested conditions. **(C)** Heatmap of differentially expressed genes clustered into four distinguished modules; the five most significant gene ontology terms for each module are presented on the right.

Our findings on TF transcription and activity indicate that hypoxia affects monocyte responses to inflammatory stimuli such as IL-1β differently depending on the specific hypoxic profile. We therefore set to study in detail the transcriptional program of primary monocytes subjected to sustained and intermittent hypoxia, with and without IL-1β stimulation, as a model for the immunothrombotic milieu in the venous valve. Overall, IL-1β alone (in normoxia) significantly changed the expression of 1430 genes, whereas exposure to sustained or intermittent hypoxia alone affected 240 and 24 genes, respectively (**Figure 3A**). A greater impact, however, was observed when monocytes subjected to hypoxic stress were stimulated with IL-1β. IL-1β stimulation in sustained hypoxia induced 1605 differentially expressed genes (DEG) in the treated monocytes in comparison to control, and 2207 DEGs in intermittent hypoxia. As depicted in **Figure 3B**, a principal component analysis showed that the most substantial transcriptomic changes in the monocytes were driven by IL-1β (PC1) and sustained hypoxia (PC2). Further, monocytes cultured in normoxia and intermittent hypoxia clustered closely together, whereas monocytes subjected to sustained hypoxia were clearly separated from these groups, both in presence and absence of IL-1β.

An overview of all differentially expressed genes in primary monocytes in the six different culture conditions is depicted in **Figure 3C**. The heatmap is divided by hierarchical clustering into four main modules. Genes included in modules one and two were primarily affected by IL-1β. Accordingly, GO term analysis of the most significant effects indicate that the genes in these modules mainly drive inflammation including cytokine signaling and inflammatory regulation. Modules three and four were predominantly driven by sustained hypoxia. GO analysis indicate that these modules mainly concern processes involved in metabolic adaptations to hypoxia, increased hydroxylation, glycolysis and GTPase activity as well as a decreased macrophage activation, and cellular processes.

In order to gain deeper insights into the impact of hypoxia on primary monocytes, we performed a gene set enrichment analysis (GSEA). The results of this analysis revealed that sustained hypoxia alone exerts a suppressive effect on transcriptional and translational machineries, as well as immune-related processes. However, it also favors proline hydroxylation and a stress response to decreased oxygen levels (**Supplementary Table 1A**). Intermittent hypoxia, on the other hand, promotes increased stress and immune responses, and interferon signaling (**Supplementary Table 1B**).

### Sustained, but not intermittent, hypoxia restricts the pro-inflammatory response to IL-1

**Figure 4:**
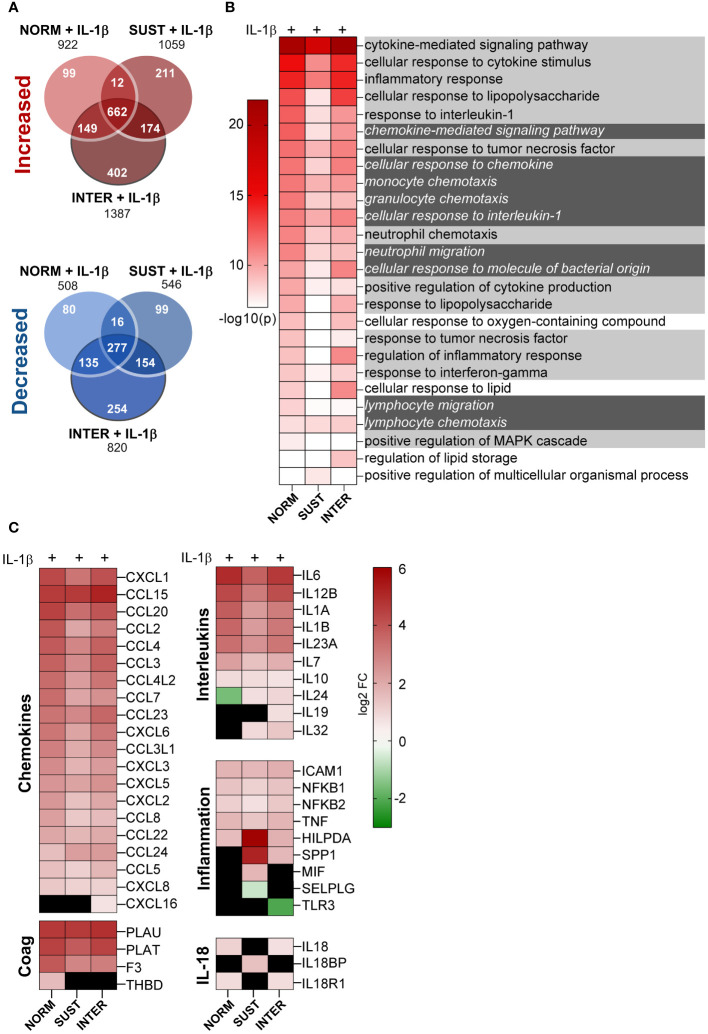
Intermittent hypoxia combined with IL-1β promotes the largest transcriptional changes and does not limit the IL-1β induced cytokine and chemokine gene responses. **(A)** The number of genes differentially increased/decreased following monocyte stimulation with IL-1β in normoxia, sustained or intermittent hypoxia in comparison to normoxia control. **(B)** GSEA-based heatmap of the 20 most significant GO terms for each IL-1β stimulation (in normoxia, sustained or intermittent hypoxia) in comparison to normoxia control. Inflammation-associated terms are marked with bright grey and chemotaxis in dark grey. **(C)** Heatmap of all differentially expressed chemokine and interleukin genes, as well as selected inflammation-related genes. Black square = non-significant difference in gene expression.

Hypoxia occurs frequently *in vivo* without inducing thrombosis. Accordingly, as described above, sustained, and intermittent hypoxia alone had modest effects on the monocyte transcriptome. However, we hypothesized that hypoxic and inflammatory stress together lower the threshold of monocytic pro-thrombotic responses, and thus promote initiation of thrombosis. Interestingly, as shown in **Figure 3A**, IL-1β combined with sustained/intermittent hypoxia induced the largest transcriptomic changes. We therefore next investigated in detail how sustained/intermittent hypoxia modulate an ongoing inflammation by comparing the transcriptomic impact of IL-1β stimulation in normoxia, sustained hypoxia and intermittent hypoxia. We observed that the majority of genes up/downregulated by IL-1β were shared by the three oxygen conditions (**Figure 4A**). Interestingly, more than 400 genes were increased and 250 were decreased only when monocytes were exposed to intermittent hypoxia combined with IL-1β (**Figure 4A**). Further, the most significantly increased biological processes (20 per condition) for monocyte responses to IL-1β in either normoxia, sustained/intermittent hypoxia revealed a high degree of similarity, as 23/26 of these GO terms concern inflammation and chemotaxis (**Figure 4B**). Sustained hypoxia, however, limited the IL-1β induction of nearly all (24/26) processes. The inflammation-limiting impact of sustained hypoxia was also observed on individual chemokine and interleukin genes, such as CCL2, IL-1β, IL-6 and IL-12 (**Figure 4C**). Intermittent hypoxia, however, did not impair the immunothrombotic effects induced by IL-1β but allowed for an unrestricted monocyte response (**Figure 4C**). A similar pattern was observed for genes involved in the plasminogen system except for VEGF which was additively increased by IL-1β combined with sustained hypoxia (**Supplementary Table 2**).

### Additive and subtractive effects of sustained and intermittent hypoxia to IL-1β stimulation

**Figure 5:**
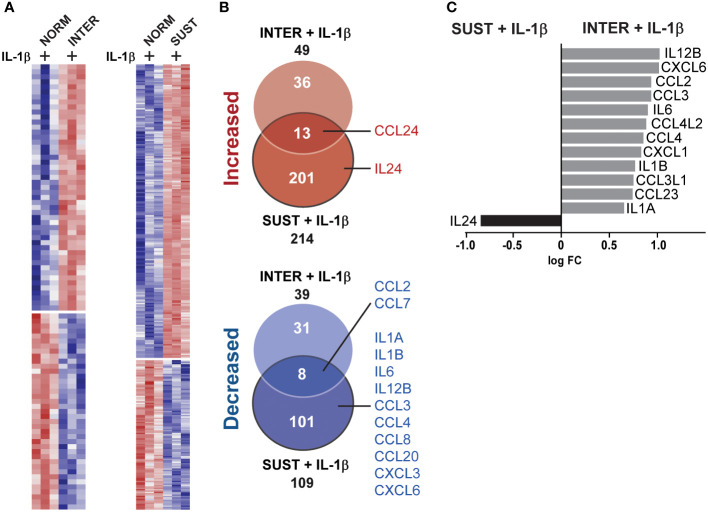
Sustained, but not intermittent, hypoxia limits optimal induction of interleukin and chemokine genes in monocytes in response to IL-1β stimulation. **(A)** A heatmap of the genes differentially expressed in monocytes stimulated with IL-1β in intermittent (left panel) or sustained hypoxia (right panel) when compared to monocytes stimulated with IL-1β in normoxia. **(B)** Venn diagrams summarizing the differentially expressed genes (up and downregulated) represented in the heatmaps presented in **(A)**, with interleukin and chemokine genes highlighted. **(C)** A direct comparison of all chemokine and interleukin genes differentially expressed following monocyte stimulation with IL-1β in sustained or intermittent hypoxia.

Whereas the combined effects of inflammatory and hypoxic stress on monocyte response are described above (**Figure 4**), we next set to identify possible additive and subtractive effects of the exposure to sustained/intermittent hypoxia on inflammatory stimulus. A comparison of the transcripts of IL-1β stimulated monocytes in normoxia with monocytes stimulated with IL-1β either in intermittent or sustained hypoxia revealed additive expression of 49 and 214 genes, respectively (**Figure 5A**). Subtractive effects were observed for 39 and 109 genes following stimulation with IL-1β in intermittent or sustained hypoxia, respectively, in comparison to monocyte stimulation with IL-1β in normoxia (**Figure 5B** and **Supplementary Table 3**). More specifically, sustained hypoxia reduced the stimulatory effect of IL-1β on key monocyte cytokines and chemokines such as IL6, IL12, CCL3 and CCL8 (**Figure 5B**). A direct comparison of the two hypoxic profiles showed that all detected chemokine and interleukin genes (apart from IL24) were expressed at higher levels in intermittent hypoxia in comparison to sustained following monocyte stimulation with IL-1β (**Figure 5C**).

### The cytokine and chemokine release from monocytes is higher in intermittent hypoxia

**Figure 6:**
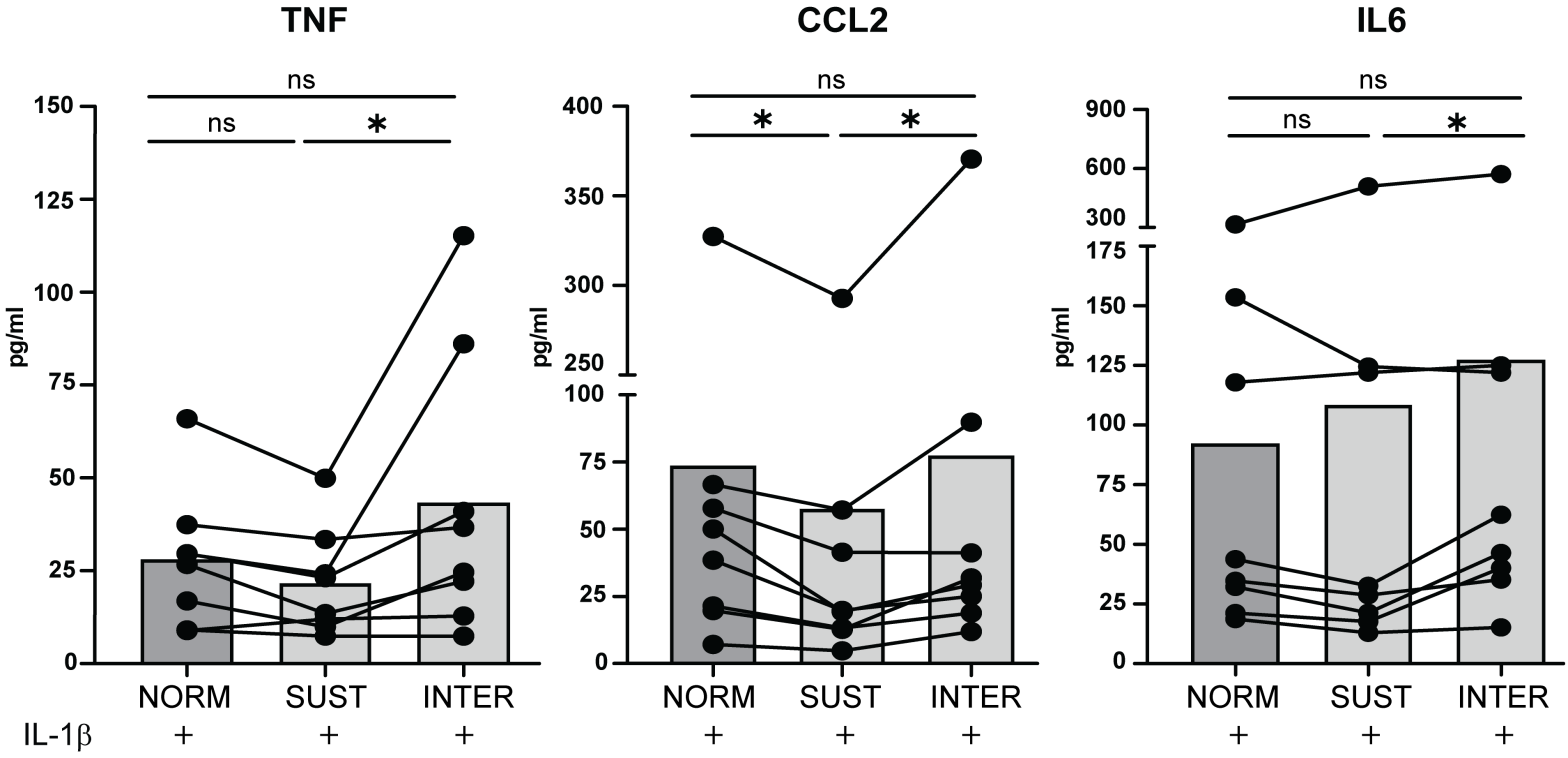
The chemokine and cytokine release from IL-1β stimulated monocytes is higher in intermittent hypoxia than in sustained hypoxia. The levels of TNF, CCL2 and IL-6 released from monocytes stimulated with IL-1β in normoxia, sustained or intermittent hypoxia were analyzed in the supernatants using ELISA. n = 8 donors distributed on 5 independent experiments. Repeated Measures one-way Anova followed by Tukey’s multiple comparison test. * p<0.05, ns = non-significant.

To validate the observed changes in the monocyte transcriptome, we measured the secretion of key monocyte chemokines and cytokines by ELISA. CCL2, TNF and IL-6 are central inflammatory mediators released by monocytes and were selected for validation on protein level in the monocyte culture supernatants. As depicted in **Figure 6**, the concentrations of CCL2, TNF and IL-6 were significantly higher in intermittent hypoxia compared to in sustained hypoxia. This aligns with the transcriptional data showing that sustained (unlike intermittent) hypoxia limited the IL-1β induced expressions of these genes. The results thus validate our transcriptome analysis, and further highlights the ability of short-term sustained hypoxia to mitigate inflammatory activation in monocytes.

## Discussion

In this work we thoroughly dissected the impact of acute sustained and intermitted hypoxia combined with inflammatory cytokine stimulation on primary monocyte responses at the transcript, protein, and functional level. We found that the two hypoxic profiles, sustained and intermittent, have greatly different impact on the monocyte transcriptome and pro-thrombotic function, both in presence and absence of IL-1β. In normoxia, IL-1β exhibited strong pro-thrombotic effects including enhanced TF expression and activity, as well as the transcription and production of pro-inflammatory interleukins and chemokines. Sustained hypoxia, however, reduced most of these effects, and instead promoted a metabolic shift in the monocyte transcriptome. Intermittent hypoxia on the other hand allowed for a complete pro-thrombotic and proinflammatory monocyte response to IL-1β. In addition, for several genes associated with VTE, the strongest induction by IL-1β stimulation depended on hypoxia.

Monocytes are key players in VTE (9), with dysregulated inflammatory cytokines playing an important role (22). Cytokines such as TNF, IL-6, IL-1β and IL-12 are associated with increased risk of VTE (23), and monocyte-derived IL-6 (4), IL-8 and CCL2 are differentially regulated during clot formation and lysis (24). Further, monocytic TF, which is a principal driver of immunothrombotic processes, is induced by such inflammatory cytokines (25–27). In line with this, we found that IL-1β alone increased the expression and activity of TF, as well as of multiple chemokine and interleukin genes and the release of IL-6, TNF and CCL2. We hypothesized that these pro-thrombotic effects of IL-1β would be modulated by hypoxia, which could exacerbate thrombotic development in the valve pocket. Monocytes experience both high and low oxygenation during their migration from bone marrow to circulation, and into inflammatory tissues (28). Sustained hypoxia alone (1%) was found by Bosco et al. to affect the expression of >1200 genes regulating glycolysis, chemotaxis, and interleukin signaling (29). It is however largely unknown how hypoxia combined with inflammatory stimulation affects primary monocyte responses. Interestingly, one of few studies on monocytes subjected to combined hypoxic/inflammatory stimulation demonstrated that hypoxia limits inflammation in sepsis through HIF-1a signaling, and instead promotes phagocytosis and tissue remodeling (30). Although highly interesting, it is unclear whether the inflammation-limiting effect was induced solely by hypoxia, as HIF-1a can also be stabilized by pathogenic stimuli including LPS (31). There is however further support for an inflammation-limiting capacity of hypoxia. Twisselman et al. found that moderate hypoxia (3% O_2_) alone had essentially no effect on monocyte-derived macrophages but, interestingly, mitigated the response to LPS and decreased the release of TNF, IL-6, and IL-1β, as well the expressions of activation markers (32).

In the current study, we used IL-1β for stimulation of monocytes, as it is more relevant than LPS for the pathogenesis of VTE. IL-1β is matured from its inactive precursor in a process catalyzed by the NLRP3 inflammasome (33). Several studies have indicated a role for the NLRP3 inflammasome in venous thrombosis (34, 35), including a central role for the combined NLRP3/IL-1β activity in VTE (36). Targeting NLRP3/IL-1β has been highlighted as a potential strategy to reduce risk or burden of VTE (37), and IL-1β has been shown to induce TF in mononuclear cells (38) and in monocytes (39). IL-1β is secreted by many different cells, however, in the venous valve pocket it is plausible that endothelial cells exposed to turbulent flow and hypoxic stress are the primary source of IL-1β. We found that sustained hypoxia effectively limited the pro-thrombotic effects of IL-1β. This was evidenced by reduced TF expression and activity, as well as attenuated induction of pro-inflammatory genes and biological processes. The paradoxical nature of hypoxia is noteworthy, and it can both promote and inhibit pro-inflammatory genes (29), and limit inflammation (40), possibly to prevent excessive inflammation-induced tissue damage (41). Additionally, sustained hypoxia can inhibit CCL2-dependent monocyte migration in an active, transcription-dependent manner (42), and reduce chemokine expressions, whereas intermittent hypoxia was shown to have the opposite effect, promoting chemokines (43). It is therefore challenging to disentangle the combined effects of hypoxia and inflammation in VTE, and it remains uncertain whether they enhance or attenuate each other’s individual effects on monocyte responses. One possible explanation for the observed inflammation-limiting effect of sustained hypoxia is the inefficient transcriptional activity owing to lower energy availability. Indeed, a major effect of sustained hypoxia in our study was the induction of a metabolic shift accompanied by reduced translation and extracellular matrix disassembly. Innate immune cells undergo such a metabolic shift to mobilize energy during hypoxic stress (44) and infection (45), which may explain the inflammation-mitigating ability of sustained hypoxia. Intriguingly, intermittent hypoxia did not limit monocyte responses to IL-1β stimulation. Instead, the combined effect of intermittent hypoxia and IL-1β treatment led to the most pronounced expression of IL-19, IL-24 and IL-32 which are known to be dysregulated in inflammatory disorders, such as atherosclerosis (46, 47).

The striking differences between the two hypoxic profiles and their impacts on immunothrombotic responses are noteworthy. We reason that in VTE, monocytes initially experience intermittent hypoxia upon entering the valvular pockets, which gradually shifts to sustained hypoxia as the thrombus grows and restricts blood flow. This sequence of events would result in unhindered inflammation and thrombogenesis in early phases, followed by a more sustained hypoxic profile with reduced inflammation in later phases. And, paradoxically, mitigated inflammation in late stages of thrombogenesis can exacerbate the situation, considering that pro-inflammatory effects support fibrinolysis. In fact, many inflammatory cues that promote thrombus growth in early phases including IL-8 and TNF also support thrombolysis (48, 49). Our observation that sustained hypoxia limits the pro-inflammatory IL-1β driven response in monocytes could, therefore, have pro-thrombotic consequences in late stages of thrombus growth. Immune cells (50) including monocytes and neutrophils play essential roles in thrombolysis through the release of inflammatory cytokines and matrix metalloproteinases (MMPs) (51). MMP-9 is central in thrombus resolution (52), and is believed to originate mainly from monocytes and monocyte-derived cells (53, 54). Furthermore, monocytes are equipped with functional PAI-1 (55), the main regulator of plasminogen activators including tPA, which in thrombi mainly stems from monocytes (56). Accordingly, we found that in normoxia, IL-1β favored potent increases in IL-8 and TNF and components of the plasminogen system, whereas sustained hypoxia mostly limited these effects. We also observed that both hypoxia profiles modulated TIMPs, proteins regulating angiogenesis and fibrinolysis by inhibiting MMPs (57). Imbalances in TIMP/MMP levels are associated with vascular disease (57) including deep vein thrombosis (58).

Although sustained hypoxia primarily limited inflammation in our study, it also demonstrated a notable ability to induce the upregulation of several thrombosis-related genes when combined with IL-1β. Among these genes, HILPDA (Hypoxia Inducible Lipid Droplet Associated) exhibited the highest upregulation. HILPDA regulates lipid storage, and is known to be induced in atherosclerotic plaques (59), where it augments atherosclerotic inflammation (60) and drives inflammation in ischemic heart disease (61). Hypoxia has been shown to induce HILPDA (62); and our study reveals an additive effect on its expression when monocytes are exposed to sustained hypoxia and IL-1β. In addition, both sustained and intermittent hypoxia inhibited the IL-1β induced expression of thrombomodulin (TM). TM is a coagulation-modulating protein expressed by endothelial cells, but also by monocytes (63), and monocytic TM potentially exerts a protective effect in cardiovascular disease (64, 65). Decreased TM expression in monocytes has been associated with poor outcome in disseminated intravascular coagulation (66). Thus, the observed inhibition of IL-1β induced TM expression in monocytes under hypoxic conditions, suggests that both sustained and intermittent hypoxia may tilt the hemostatic balance towards thrombotic development. The pro-inflammatory and chemotactic macrophage inhibition factor (MIF), known to be induced by hypoxia (67), was upregulated only when sustained hypoxia was combined with IL-1β in our study. MIF can be released from activated monocytes (67), and promote the activation of NLRP3 inflammasomes (42), which is associated with cardiovascular disease^43.^ Inflammasome activation leads to the production of mature IL-1β and IL-18, with IL-18 being regulated by the soluble IL-18 binding protein (IL-18BP) (68). Interestingly, in normoxia and intermittent hypoxia, IL-1β promoted both IL-18 and its receptor IL18R. However, in sustained hypoxia, IL-1β had the opposite effect on the IL-18 system, promoting only IL18BP. This, combined with the promotion of MIF, indicates a hypoxia-driven inflammasome dysregulation.

Additionally, our study identified SPP1 as a major target gene for hypoxic modulation in monocytes. SPP1, also known as osteopontin, was one of the most significantly increased genes in hypoxic conditions, both with and without IL-1β. Osteopontin is associated with cardiovascular diseases including myocardial infarction, diabetes and atherosclerosis (69). A recent study highlighted SPP1 as a central mediator of monocyte-to-endothelial signaling in thrombosis (70), and elevated osteopontin is a strong predictor of poor outcome in stroke, myocardial infarction (71) and DVT (72). Hypoxia induces osteopontin (73), and we here provide evidence for an additive effect of hypoxia combined with inflammation on gene expression of osteopontin. Furthermore, Osteopontin can be cleaved by thrombin and MMPs into isoforms associated with more severe vascular pathology (71) increased inflammation in carotid plaques (74) and cardiac fibrosis (75). Therefore, it can be speculated that a combination of inflammatory and hypoxic stimulation exacerbates the pro-thrombotic process through monocyte-released osteopontin.

It is of importance to note that the current study investigated the effects of short-term acute hypoxia to model the very early events in VTE. Longer periods of either sustained or intermittent hypoxia may have a more pronounced (or different) impact on the function of monocytes. Chronic hypoxia leads to more buildup of hypoxia-responsive transcription factors including HIF-1a, and generates reactive oxygen species (ROS) which are strongly pro-inflammatory and may further aggravate pro-thrombotic processes (15).

## Conclusions and future suggestions

Our findings suggest that the hypoxic profile is pivotal for the immunothrombotic response in monocytes secondary to inflammatory stimulation. This may offer new perspectives on the cellular processes in the venous valve pocket, which ultimately lead to the formation of venous thrombi. As our study focused solely on the effects of short-term hypoxic and inflammatory stress on monocytes, future studies should expand the scope and investigate longer stimulations and the complex interplay between monocytes and other intravascular cells in the hypoxic valve pocket. In addition, it would be highly interesting to evaluate both the underlying mechanisms of how hypoxia mitigates the stimulatory effects of IL-1β on monocytes, and whether this effect is specific for IL-1β. Together, these studies will shed light on the cellular and molecular mechanisms that trigger and drive VTE and may thus identify novel biomarkers and targets for therapeutic intervention.

## Data availability statement

The datasets presented in this study can be found in online repositories. The names of the repository/repositories and accession number(s) can be found below: GSE234701 (GEO).

## Ethics statement

The studies involving humans were approved by The Regional Committee for Medical Research Ethics Northern Norway (REK). The studies were conducted in accordance with the local legislation and institutional requirements. The participants provided their written informed consent to participate in this study.

## Author contributions

The study was conceived by CW, J-BH, OS, experiments were designed and conducted by CW, SC, PC, data was analyzed by CW, SC, PC and interpreted by all authors, the manuscript was drafted by CW and all authors contributed to its completion and approved its final version.

## Supplementary material

Supplementary Table 1 Top 20 Biological processes induced by sustained or intermittent hypoxia in control conditions (without IL-1β) as determined by gene set enrichment analysis. GO terms decreased in the test condition compared to the reference are highlighted in grey. NES: Normalized enrichment score**. (A)** *Effects of sustained hypoxia alone on monocytes in comparison to normoxia controlB) Effects of intermittent hypoxia alone on monocytes in comparison to normoxia control.*

Supplementary Table 2 The effects of IL-1β stimulation in normoxia or sustained/intermittent hypoxia on genes related to the plasminogen system and fibrinolysis. All fold changes are in comparison to Normoxia CTRL.

Supplementary Table 3 Additive/subtractive effects of combining IL-1β with hypoxia. **(A)** 150 most significantly changed genes in Sust hypox IL-1β (fold changes to Normoxia IL-1β) **(B)** All significantly changed genes (88 detected) in INTER hypox IL-1β (changes to NORMOX IL-1β).
