# Supplementary material for: Sustained and intermittent hypoxia differentially modulate primary monocyte immunothrombotic responses to IL-1β stimulation
Source: Front Immunol. 2023 Sep 11;14:1240597. doi: 10.3389/fimmu.2023.1240597 (PMC10518394; doi:10.3389/fimmu.2023.1240597)
Supplement: Supplementary Table 1 — Top 20 Biological processes induced by sustained or intermittent hypoxia in control conditions (without IL-1β) as determined by gene set enrichment analysis. GO terms decreased in the test condition compared to the reference are highlighted in grey. NES: Normalized enrichment score. (A) Effects of sustained hypoxia alone on monocytes in comparison to normoxia controlB) Effects of intermittent hypoxia alone on monocytes in comparison to normoxia control. [file Table_1.docx]

**Supplementary Table 1**

Top 20 Biological processes induced by sustained or intermittent hypoxia in control conditions (without IL-1β) as determined by gene set enrichment analysis.

GO terms decreased in the test condition compared to the reference are highlighted in grey. NES: Normalized enrichment score.

1. *Effects of sustained hypoxia alone* *on monocytes in comparison to normoxia control*

| **Pathway** | **-log10(p)** | **NES** |
| --- | --- | --- |
| Defense response to symbiont | 7.0 | 2.2 |
| Defense response to virus | 7.0 | 2.2 |
| Translation | 5.8 | -1.9 |
| SRP-dependent cotranslational protein targeting to membrane | 5.2 | -2.0 |
| Cellular response to decreased oxygen levels | 4.8 | 2.3 |
| Cotranslational protein targeting to membrane | 5.0 | -2.0 |
| Cytoplasmic translation | 4.7 | -1.9 |
| Extracellular matrix disassembly | 4.8 | -2.1 |
| Neutrophil degranulation | 4.8 | -1.6 |
| Neutrophil mediated immunity | 4.7 | -1.6 |
| Nuclear-transcribed mrna catabolic process, nonsense-mediated decay | 4.7 | -1.8 |
| Protein targeting to ER | 4.9 | -1.9 |
| Neutrophil activation involved in immune response | 4.5 | -1.6 |
| Cellular response to hypoxia | 4.4 | 2.0 |
| Rrna processing | 4.2 | -1.7 |
| Rrna metabolic process | 4.1 | -1.8 |
| Peptidyl-proline hydroxylation to 4-hydroxy-L-proline | 4.0 | 2.0 |
| Peptide biosynthetic process | 3.7 | -1.7 |
| Peptidyl-proline hydroxylation | 3.7 | 2.0 |
| Positive regulation of cold-induced thermogenesis | 3.7 | 2.0 |

1. *Effects of intermittent hypoxia alone on monocytes in comparison to normoxia control*

| **Pathway** | **-log10(p)** | **NES** |
| --- | --- | --- |
| Defense response to virus | 8.9 | 2.2 |
| Defense response to symbiont | 8.3 | 2.2 |
| Cellular response to type I interferon | 8.0 | 2.3 |
| Type I interferon signaling pathway | 8.0 | 2.3 |
| Negative regulation of viral genome replication | 6.9 | 2.2 |
| Regulation of viral genome replication | 5.8 | 2.1 |
| Negative regulation of viral process | 5.0 | 2.0 |
| Astrocyte differentiation | 4.4 | -1.9 |
| Defense response to bacterium | 4.1 | 1.9 |
| Aerobic electron transport chain | 4.0 | 1.9 |
| Cotranslational protein targeting to membrane | 4.0 | 1.8 |
| Positive regulation of epithelial cell differentiation | 3.9 | 1.9 |
| Innate immune response | 3.9 | 1.7 |
| Protein targeting to ER | 3.9 | 1.8 |
| Nuclear-transcribed mrna catabolic process, nonsense-mediated decay | 3.9 | 1.8 |
| Mitochondrial ATP synthesis coupled electron transport | 3.9 | 1.9 |
| SRP-dependent cotranslational protein targeting to membrane | 3.8 | 1.9 |
| Cytokine-mediated signaling pathway | 3.8 | 1.5 |
| Defense response to Gram-negative bacterium | 3.7 | 1.9 |
| Vitamin K metabolic process | 3.4 | 1.8 |
