# Supplementary material for: Sustained and intermittent hypoxia differentially modulate primary monocyte immunothrombotic responses to IL-1β stimulation
Source: Front Immunol. 2023 Sep 11;14:1240597. doi: 10.3389/fimmu.2023.1240597 (PMC10518394; doi:10.3389/fimmu.2023.1240597)
Supplement: Supplementary Table 2 — The effects of IL-1β stimulation in normoxia or sustained/intermittent hypoxia on genes related to the plasminogen system and fibrinolysis. All fold changes are in comparison to Normoxia CTRL. [file Table_2.docx]

**Supplementary Table 2**

The effects of IL-1β stimulation in normoxia or sustained/intermittent hypoxia on genes related to the plasminogen system and fibrinolysis. All fold changes are in comparison to Normoxia CTRL.

|  | **NORMOX IL1b** | | **SUST IL1b** | | **INTER IL1b** | |
| --- | --- | --- | --- | --- | --- | --- |
| **Gene** | **log FC** | **-log10(p)** | **log FC** | **-log10(p)** | **log FC** | **-log10(p)** |
| PLAT | 3.7 | 5.1 | 3.2 | 5.2 | 3.7 | 6.0 |
| PLAU | 3.9 | 4.4 | 3.9 | 5.4 | 4.0 | 5.7 |
| PLAUR | 1.4 | 3.5 | 1.0 | 5.4 | 1.5 |  |
| SERPINE1 | 0.8 | 2.6 | 1.0 | 4.1 | 1.2 | 5.0 |
| VEGFA | 0.6 | 2.6 | 1.6 | 6.9 | 0.9 | 5.0 |
| VEGFB |  |  | 0.9 | 2.2 |  |  |
| TGFB2 | -1.7 | 2.5 | -1.5 | 3.4 | -1.9 | 3.6 |
| MMP2 |  |  | -1.2 | 4.3 | -0.7 | 2.4 |
| MMP9 | 2.1 | 4.3 | 1.5 | 3.7 | 2.6 | 5.8 |
| MMP14 | 2.9 | 8.0 | 2.2 | 8.7 | 3.2 | 8.7 |
| MMP17 |  |  |  |  | 0.8 | 1.5 |
| MMP19 | 0.7 | 2.5 | 0.8 | 4.2 | 0.9 | 4.1 |
| MMP25 |  |  |  |  | 0.5 | 0.8 |
| TIMP1 |  |  | 0.7 | 2.7 | 1.2 | 6.0 |
| TIMP2 |  |  | -0.8 | 4.3 | -0.7 | 5.7 |
| FGF11 |  |  | 2.9 | 3.2 |  |  |
| HBEGF |  |  | -0.8 | 3.9 | -0.8 | 3.7 |
| PDGFA | 2.6 | 4.0 |  |  | 2.2 | 4.4 |
| PDGFB | 1.5 | 5.0 | 1.1 | 5.6 | 1.6 | 5.9 |
| PDGFRB | 1.8 | 3.7 | 1.4 | 5.2 | 1.7 | 4.0 |
| THBS1 | 1.7 | 4.5 | 1.4 | 4.9 | 1.6 | 4.9 |
