# Supplementary material for: Sustained and intermittent hypoxia differentially modulate primary monocyte immunothrombotic responses to IL-1β stimulation
Source: Front Immunol. 2023 Sep 11;14:1240597. doi: 10.3389/fimmu.2023.1240597 (PMC10518394; doi:10.3389/fimmu.2023.1240597)
Supplement: Supplementary Table 3 — Additive/subtractive effects of combining IL-1β with hypoxia. (A) 150 most significantly changed genes in Sust hypox IL-1β (fold changes to Normoxia IL-1β) (B) All significantly changed genes (88 detected) in INTER hypox IL-1β (changes to NORMOX IL-1β). [file Table_3.docx]

**Supplementary Table 3** Additive/subtractive effects of combining IL-1β with hypoxia.

1. 150 most significantly changed genes in Sust hypox IL-1β (fold changes to Normoxia IL-1β)

| **Gene** | **Log FC** | **-10 log(p)** | **Gene** | **Log FC** | **-10 log(p)** | **Gene** | **Log FC** | **-10 log(p)** |
| --- | --- | --- | --- | --- | --- | --- | --- | --- |
| HILPDA | 4.8 | 9.5 | CD93 | -0.7 | 4.1 | ANKZF1 | 0.7 | 3.2 |
| LDHA | 2.9 | 9.0 | UAP1L1 | 1.1 | 4.0 | FAM234B | 0.7 | 3.1 |
| SPP1 | 5.0 | 8.8 | EHD1 | -0.9 | 4.0 | TRMT6 | -0.7 | 3.1 |
| VGLL4 | 3.0 | 8.7 | XBP1 | -0.9 | 4.0 | RNMT | 0.9 | 3.1 |
| BNIP3 | 3.2 | 8.6 | FOSL2 | 0.7 | 4.0 | ACBD5 | 0.6 | 3.1 |
| HK2 | 2.5 | 8.5 | DDIT4 | 0.9 | 3.9 | GPR84 | -0.7 | 3.1 |
| TCAF2 | 2.8 | 8.1 | CCL3 | -0.9 | 3.9 | ALOX5AP | -0.6 | 3.1 |
| SLC2A3 | 1.9 | 7.6 | CCL4 | -1.0 | 3.9 | LGMN | -0.6 | 3.1 |
| NFXL1 | 1.9 | 7.5 | PDK3 | 0.8 | 3.9 | ZCCHC2 | 0.6 | 3.1 |
| P4HA1 | 1.7 | 7.5 | TENT5A | 0.8 | 3.8 | PFKFB4 | 0.8 | 3.1 |
| BNIP3L | 1.7 | 7.4 | FAM162A | 1.5 | 3.8 | AVIL | 0.6 | 3.0 |
| KDM3A | 1.8 | 7.2 | HEG1 | 0.8 | 3.8 | LYPD3 | -1.3 | 3.0 |
| ZNF395 | 2.0 | 6.9 | CP | 3.7 | 3.7 | CCL3L1 | -1.0 | 3.0 |
| PLIN2 | 1.7 | 6.7 | CCL4L2 | -1.1 | 3.7 | HSD17B4 | 0.6 | 3.0 |
| LEP | 4.4 | 6.5 | MAF | -0.9 | 3.7 | PDE3B | 0.6 | 3.0 |
| FAM210A | 1.6 | 6.0 | IL18BP | 1.0 | 3.7 | SLC25A3 | -0.6 | 3.0 |
| LGALS8 | 1.6 | 6.0 | MAP3K7CL | 0.7 | 3.7 | PTPRJ | 0.7 | 3.0 |
| EGLN1 | 1.7 | 5.8 | PFKP | 0.8 | 3.7 | MCUB | -0.6 | 3.0 |
| GPI | 1.9 | 5.8 | ENO2 | 2.0 | 3.6 | BYSL | -1.1 | 2.9 |
| ZNF160 | 1.3 | 5.7 | TFRC | 0.8 | 3.6 | CXCL1 | -1.0 | 2.9 |
| ERO1A | 1.1 | 5.7 | IL1B | -1.2 | 3.6 | RAPGEF6 | 0.8 | 2.9 |
| SFXN1 | 1.9 | 5.7 | P4HA2 | 1.6 | 3.6 | FABP3 | 1.8 | 2.9 |
| YEATS2 | 1.3 | 5.6 | PHF13 | 0.7 | 3.6 | TCEAL9 | 0.8 | 2.9 |
| ZNF292 | 2.3 | 5.6 | CREBRF | 0.7 | 3.6 | IL12B | -1.1 | 2.9 |
| PKD2 | 1.4 | 5.4 | CD180 | 0.7 | 3.5 |  |  |  |
| MARCHF1 | 1.0 | 5.4 | CXCL2 | -0.9 | 3.5 |  |  |  |
| ANKRD37 | 3.2 | 5.3 | ARL10 | 2.0 | 3.5 |  |  |  |
| NGLY1 | 1.1 | 5.2 | IL24 | 1.6 | 3.5 |  |  |  |
| MTFP1 | 3.4 | 5.2 | TKTL1 | 1.9 | 3.5 |  |  |  |
| NARF | 1.3 | 5.1 | HDHD5 | 1.5 | 3.5 |  |  |  |
| SEMA4B | 1.4 | 5.1 | ECE1 | -0.6 | 3.5 |  |  |  |
| ZNF654 | 1.1 | 5.0 | FCGR2B | 1.3 | 3.4 |  |  |  |
| PDK1 | 1.6 | 4.9 | YARS1 | -0.7 | 3.4 |  |  |  |
| SNAPC1 | 1.3 | 4.9 | SLC23A2 | 0.7 | 3.4 |  |  |  |
| SLC2A1 | 1.5 | 4.8 | PIGA | 1.0 | 3.4 |  |  |  |
| DARS1 | 0.9 | 4.8 | ZBTB25 | 0.9 | 3.4 |  |  |  |
| CXCR4 | 1.4 | 4.8 | ALOX5 | -0.9 | 3.4 |  |  |  |
| NIPAL1 | 1.7 | 4.8 | DGKE | 0.8 | 3.4 |  |  |  |
| FAM13A | 1.3 | 4.7 | MMP14 | -0.7 | 3.4 |  |  |  |
| CCL2 | -1.8 | 4.7 | IL1A | -1.4 | 3.4 |  |  |  |
| IL6 | -1.2 | 4.5 | KLHL24 | 0.7 | 3.4 |  |  |  |
| PLOD2 | 2.5 | 4.5 | TNFRSF10D | 0.7 | 3.4 |  |  |  |
| UFSP2 | 1.0 | 4.4 | ABCC1 | 0.6 | 3.4 |  |  |  |
| ACP5 | -1.6 | 4.4 | MXI1 | 0.9 | 3.3 |  |  |  |
| EGLN3 | 2.3 | 4.4 | ARHGEF26 | 1.7 | 3.3 |  |  |  |
| RLF | 1.0 | 4.4 | TTC7A | -0.7 | 3.3 |  |  |  |
| CCL24 | 0.8 | 4.4 | PPP1R3E | 0.8 | 3.3 |  |  |  |
| VLDLR | 1.9 | 4.4 | MNT | 0.7 | 3.3 |  |  |  |
| C4orf3 | 0.8 | 4.3 | FRRS1 | 1.1 | 3.3 |  |  |  |
| CD300A | 1.7 | 4.3 | IPMK | 0.6 | 3.3 |  |  |  |
| ZBTB1 | 0.8 | 4.3 | HSPA5 | -0.6 | 3.3 |  |  |  |
| KLF9 | 0.8 | 4.3 | OXSM | 1.3 | 3.3 |  |  |  |
| PTX3 | -0.8 | 4.2 | USP37 | 0.7 | 3.3 |  |  |  |
| TNS1 | 0.8 | 4.2 | GYS1 | 0.8 | 3.3 |  |  |  |
| FBP1 | -1.7 | 4.2 | TM4SF19 | 1.0 | 3.3 |  |  |  |
| LZTS2 | 1.6 | 4.2 | RHOBTB2 | 1.1 | 3.2 |  |  |  |
| BHLHE40 | 1.0 | 4.1 | SPTSSA | 0.6 | 3.2 |  |  |  |
| VEGFA | 1.0 | 4.1 | RNF217 | 0.7 | 3.2 |  |  |  |
| CCNG2 | 0.7 | 4.1 | F13A1 | 0.7 | 3.2 |  |  |  |
| HIF1A | -0.7 | 4.1 | FUT11 | 0.8 | 3.2 |  |  |  |
| MYC | -1.1 | 4.1 | RNF24 | 0.6 | 3.2 |  |  |  |
| CPVL | -0.7 | 4.1 | PNO1 | -0.7 | 3.2 |  |  |  |
| GCLC | 0.7 | 4.1 | CCL7 | -1.3 | 3.2 |  |  |  |

1. All significantly changed genes (88 detected) in INTER hypox IL-1β (changes to NORMOX IL-1β)

| **Gene** | **Log FC** | **-10 log p** | **Gene** | **Log FC** | **-10 log p** |
| --- | --- | --- | --- | --- | --- |
| NFXL1 | 0.8 | 4.8 | NPIPB6 | -2.5 | 2.3 |
| VGLL4 | 1.1 | 4.3 | OSBPL1A | -1.0 | 2.3 |
| THBD | -1.5 | 3.4 | APBB2 | -1.5 | 2.2 |
| SLX1B | 2.9 | 3.4 | NPIPB9 | 1.2 | 2.2 |
| ASNS | -0.7 | 3.4 | CXorf65 | -1.3 | 2.2 |
| SELL | -0.6 | 3.3 | BBC3 | 0.7 | 2.2 |
| SCP2 | -0.6 | 3.0 | TUBE1 | -0.7 | 2.2 |
| CLEC12B | -0.8 | 2.8 | GPRASP1 | -1.0 | 2.2 |
| LRRC69 | -1.8 | 2.7 | ADGRB1 | 0.9 | 2.2 |
| CTSO | -0.6 | 2.7 | LHX4 | -1.2 | 2.2 |
| ERMN | -0.7 | 2.7 | TBC1D8B | -1.5 | 2.2 |
| CCL24 | 0.9 | 2.7 | MICALL2 | 0.6 | 2.2 |
| SEZ6L | -1.7 | 2.7 | EFCAB2 | -0.8 | 2.2 |
| CSKMT | 0.9 | 2.7 | APOC2 | 1.6 | 2.2 |
| GGTA1 | -1.2 | 2.6 | INF2 | 0.6 | 2.2 |
| CCL2 | -0.9 | 2.6 | KCNJ11 | 0.6 | 2.1 |
| CD69 | -0.6 | 2.6 | BAIAP2 | 0.8 | 2.1 |
| MVD | 0.6 | 2.6 | SNURF | 1.4 | 2.1 |
| FBP1 | -1.0 | 2.6 | FCGBP | 1.2 | 2.1 |
| TSEN15 | -0.6 | 2.6 | TNFRSF4 | 1.3 | 2.1 |
| RHOBTB2 | 1.0 | 2.6 | FTL | 0.7 | 2.1 |
| ANAPC10 | -0.6 | 2.6 | SEMA4B | 0.6 | 2.1 |
| NUMBL | 1.0 | 2.5 | PTGS2 | -0.8 | 2.1 |
| SPP1 | 1.2 | 2.5 | TMEM150B | 0.7 | 2.1 |
| ROGDI | 0.9 | 2.5 | PHEX | -0.9 | 2.1 |
| HR | 0.9 | 2.5 | APBB1 | 1.9 | 2.1 |
| UBA5 | -0.7 | 2.5 | PRUNE2 | -1.5 | 2.1 |
| BNIP3 | 0.6 | 2.5 | MIB2 | 0.6 | 2.1 |
| ZNF444 | 0.8 | 2.5 | BBS1 | 0.6 | 2.0 |
| BRMS1L | -0.7 | 2.5 | IDNK | -0.9 | 2.0 |
| CCL7 | -0.8 | 2.5 | L1CAM | 1.4 | 2.0 |
| NOL3 | 0.7 | 2.5 | ZNF366 | 0.8 | 2.0 |
| SGSH | 0.6 | 2.5 | CCDC170 | -1.2 | 2.0 |
| PINLYP | 0.9 | 2.4 | OLFM2 | 1.2 | 2.0 |
| H2BC4 | 1.1 | 2.4 | DAGLA | 0.8 | 2.0 |
| ZFP28 | -0.8 | 2.4 | UROS | -0.7 | 2.0 |
| H2BC12 | 1.0 | 2.4 | NPIPB6 | -2.5 | 2.3 |
| NOTCH3 | 0.6 | 2.4 | OSBPL1A | -1.0 | 2.3 |
| HAUS7 | 0.6 | 2.4 | APBB2 | -1.5 | 2.2 |
| FAM161A | -1.5 | 2.4 | NPIPB9 | 1.2 | 2.2 |
| TMEM184B | 0.6 | 2.4 | CXorf65 | -1.3 | 2.2 |
| FITM1 | 1.5 | 2.4 | BBC3 | 0.7 | 2.2 |
| PLXNA3 | 0.6 | 2.4 | TUBE1 | -0.7 | 2.2 |
| GGT1 | 0.7 | 2.4 | GPRASP1 | -1.0 | 2.2 |
| MAP2K7 | 0.6 | 2.4 | ADGRB1 | 0.9 | 2.2 |
| PRDM15 | 0.7 | 2.3 | LHX4 | -1.2 | 2.2 |
| SCIMP | -0.7 | 2.3 | TBC1D8B | -1.5 | 2.2 |
| B9D2 | 1.1 | 2.3 | MICALL2 | 0.6 | 2.2 |
| TUFT1 | -0.6 | 2.3 | EFCAB2 | -0.8 | 2.2 |
| MSH2 | -0.9 | 2.3 | APOC2 | 1.6 | 2.2 |
| ZNF292 | 0.9 | 2.3 | INF2 | 0.6 | 2.2 |
| TNFRSF21 | -0.7 | 2.3 | KCNJ11 | 0.6 | 2.1 |
